# Plant ISOform sequencing database (PISO): a comprehensive repertory of full‐length transcripts in plants

**DOI:** 10.1111/pbi.13076

**Published:** 2019-01-28

**Authors:** Jia‐Wu Feng, Shanshan Huang, Yi‐Xiong Guo, Dongxu Liu, Jia‐Ming Song, Junxiang Gao, Huan Li, Ling‐Ling Chen

**Affiliations:** ^1^ National Key Laboratory of Crop Genetic Improvement Huazhong Agricultural University Wuhan China; ^2^ Hubei Key Laboratory of Agricultural Bioinformatics College of Informatics Huazhong Agricultural University Wuhan China

**Keywords:** alternative splicing, alternative polyadenylation, full‐length transcripts, isoform sequencing

Dear Editor,

In higher eukaryotes, alternative splicing (AS) and alternative polyadenylation (APA) events can produce multiple transcript isoforms in the majority of genes, which significantly increase the protein‐coding potential of a genome (Anvar *et al*., 2018; Pan *et al*., 2008). Different transcript isoforms might encode proteins with different functions or affect the mRNA stability and translational capacity, in some sense AS and APA events can dramatically increase the complexity and flexibility of the entire transcriptome and proteome (Feng *et al*., 2015; Li *et al*., 2017a; Wang *et al*., 2017a; Yang *et al*., 2016). Many databases contained AS events and transcripts in animals are available in some public resources such as ASTD and MAASE (Zheng *et al*., 2005), whereas there is no database containing full‐length transcripts and AS events in plants up to now. Next‐generation sequencing (NGS) technology has limitation for identifying AS and APA events due to short reads and low accuracy. In recent years, isoform sequencing (Iso‐Seq) using Pacbio single molecule real‐time sequencing (SMRT) platform can generate full‐length sequences and provide accurate information about AS and transcriptional start sites (Li *et al*., 2017a). In this study, we collected the plant Iso‐Seq data sequenced by Pacbio platform from NCBI database up to the end of 2017, and employed unified pipelines to process all the full‐length transcripts in different species. Based on these data, we constructed Plant ISOform sequencing database (PISO, http://cbi.hzau.edu.cn/piso/).

**Figure 1:**
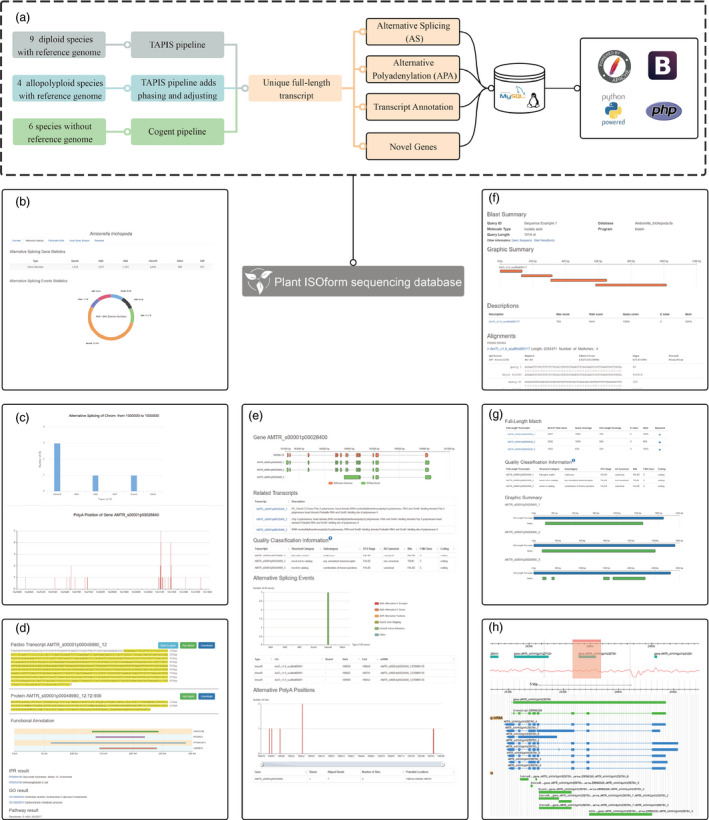
The architecture, pipeline and some screenshots in PISO. (a) The architecture and pipeline of PISO. (b) Species homepage. (c) Alternative splicing search. (d) Transcript browser results for one transcript. (e) Transcript browser results for one gene. (f) BLAST results. (g) Full‐length match results. (h) GBrwose.

Plant ISOform sequencing database was performed on Linux operation system and Apache web server (http://www.apache.org/). The obtained transcripts, AS events, novel genes and analysis tools were organized and stored in MySQL database (http://www.mysql.com/). Bootstrap framework (https://getbootstrap.com/) and jQuery (https://blog.jquery.com/) were applied for constructing the website, and PHP was used to interact with back‐end data and ECharts (http://echarts.baidu.com/)/Highcharts (http://www.hcharts.cn/) for data visualization. Currently, there are 19 plant species listed in PISO (i.e. *Amborella trichopoda, Arabidopsis thaliana, Beta vulgaris* subsp. *Vulgaris, Chenopodium quinoa, Coffea arabica, Fragaria vesca, Gossypium barbadense, Hevea brasiliensis, Panax ginseng, Phyllostachys edulis, Sorghum bicolor, Triticum aestivum, Zea mays, Allium sativum, Astragalus membranaceus, Dipteryx oleifera, Nepenthes ampullaria, Nepenthes rafflesiana* and *Salvia miltiorrhiza*), of which 13 have reference genomes (nine diploids and four allopolyploids), while the other six species do not have genome sequences. We employed three pipelines for analysing Iso‐Seq data of the above three types of plant species (Figure 1a). Firstly, standard TAPIS pipeline was utilized for nine diploids with reference genomes (Abdelghany *et al*., 2016; Wang *et al*., 2017a); secondly, two extra steps, phasing and adjusting, were added into TAPIS pipeline for studying the four allopolyploid species owing to the large number of homoeologous genes from different sub‐genomes (Wang *et al*., 2017b); thirdly, for analysing the other six species without reference genome, high‐quality reads were mapped to a pseudo‐genome generated by Cogent software (Li *et al*., 2017b).

Plant ISOform sequencing database provided millions of high quality transcript isoforms. In total, 1 391 165 transcripts, 50 803 novel gene loci, 878 057 AS and 81 416 APA events were obtained from 19 plant species. Different types of AS events, i.e. intron retention (IntronR), exon skipping (ExonS), alternative donor site (AltD), alternative acceptor site (AltP), alternative position (AltP) and other types of AS were provided for each species. For instance, the genome of *Amborella trichopoda* in Ensembl Plants database contains 27 313 protein‐coding gene loci and only 27 313 transcripts. In PISO, 34 733 transcripts, 15 039 AS events and 3315 APA events were obtained for the detected 9060 expressed protein‐coding gene loci, and 769 novel gene loci were identified and listed. Therefore, transcripts and novel protein‐coding gene loci gained in PISO greatly expanded the current genome annotation. Simultaneously, PISO provided functional annotation and protein sequence for each transcript.

Plant ISOform sequencing database has a flexible user interface. A great number of interactive graphs generated by JavaScript were used to display significant results. For example, the doughnut chart illustrated the distribution of different types of AS events in each species (Figure 1b). Meanwhile, AS statistics, GO statistics and browser of novel genes were integrated in ‘Species’ page. Locations on chromosome and gene names can be used to identify the AS and APA events on ‘Alternative Splicing’ page (Figure 1c). Several bar charts demonstrated the statistics of APA positions and AS events for each gene. Besides, the customized graphs showed the relative positions of different sequences by using tools of ‘Functional Annotation’, BLAST, ‘Full‐length Match’ and ‘Transcript Browser’. Protein functional annotation linked to other databases and the sequence of each transcript for a given gene was displayed in Figure 1d. Users can submit a gene name on ‘Transcript Browser’ page, and the server will return the information of AS events, APA events and transcripts of this gene. At the same time, PISO also supplied annotation corresponding to different transcripts (Figure 1e).

Several convenient tools were provided in PISO, such as Functional Search, BLAST, Full‐length Match and GBrowse. In ‘Functional Search’ section, transcripts can be retrieved by InterPro accession, GO ID, Pfam accession, transcript name, gene name, etc., the description of different transcripts can also be exhibited in this page. After selecting sequence and parameters, BLAST would return detailed results (Figure 1f). Moreover, PISO provided a useful tool, Full‐Length Match, to search for the best full‐length transcript that matched the user‐uploaded sequence (Figure 1g). Based on a new sequence, gene name or transcript name can be obtained through Blast or ‘Full‐Length Match’. A local GBrowse offered user with manipulating and displaying annotation on genomes. Detailed information of specific regions on the chromosome can be accessed by GBrowse (Figure 1h).

In summary, we constructed a significant bioinformatics platform, PISO, to provide a comprehensive repertory of sequenced Iso‐Seq full‐length transcripts for 19 plant species. PISO possesses a flexible user interface to display transcripts, novel genes, AS and APA events. Furthermore, it involves the pipeline, guide document and reference links. With the widespread application of Pacbio SMRT‐based sequencing, PISO will be continuously updated and provide more valuable information for researchers. PISO is freely available at http://cbi.hzau.edu.cn/piso/.
